# Semi-automated left ventricular segmentation based on a guide point model approach for 3D cine DENSE cardiovascular magnetic resonance

**DOI:** 10.1186/1532-429X-16-8

**Published:** 2014-01-14

**Authors:** Daniel A Auger, Xiaodong Zhong, Frederick H Epstein, Ernesta M Meintjes, Bruce S Spottiswoode

**Affiliations:** 1 MRC/UCT Medical Imaging Research Unit, Department of Human Biology, University of Cape Town, Cape Town, South Africa; 2 MR R&D Collaborations, Siemens Medical Solutions, Atlanta, GA, USA; 3 Departments of Radiology and Biomedical Engineering, University of Virginia, Charlottesville, VA, USA; 4 Cardiovascular MR R&D, Siemens Healthcare, Chicago, IL 60611, USA

**Keywords:** Cardiovascular MR, DENSE, Segmentation, Guide point modeling

## Abstract

**Background:**

The most time consuming and limiting step in three dimensional (3D) cine displacement encoding with stimulated echoes (DENSE) MR image analysis is the demarcation of the left ventricle (LV) from its surrounding anatomical structures. The aim of this study is to implement a semi-automated segmentation algorithm for 3D cine DENSE CMR using a guide point model approach.

**Methods:**

A 3D mathematical model is fitted to guide points which were interactively placed along the LV borders at a single time frame. An algorithm is presented to robustly propagate LV epicardial and endocardial surfaces of the model using the displacement information encoded in the phase images of DENSE data. The accuracy, precision and efficiency of the algorithm are tested.

**Results:**

The model-defined contours show good accuracy when compared to the corresponding manually defined contours as similarity coefficients Dice and Jaccard consist of values above 0.7, while false positive and false negative measures show low percentage values. This is based on a measure of segmentation error on intra- and inter-observer spatial overlap variability. The segmentation algorithm offers a 10-fold reduction in the time required to identify LV epicardial and endocardial borders for a single 3D DENSE data set.

**Conclusion:**

A semi-automated segmentation method has been developed for 3D cine DENSE CMR. The algorithm allows for contouring of the first cardiac frame where blood-myocardium contrast is almost nonexistent and reduces the time required to segment a 3D DENSE data set significantly.

## Background

Cardiovascular magnetic resonance (CMR) provides accurate and reproducible quantitative measurements of cardiac functional parameters for the diagnosis and treatment of cardiovascular disease. Techniques include balanced steady state free precession (SSFP) for morphological cine imaging [1,2], myocardial tagging for intra-myocardial strain analysis [3,4] and phase contrast velocity encoding for tissue velocity and strain rate imaging [5,6]. Each technique plays an important role in quantifying myocardial function, however, each consists of inherent limitations. Myocardial tagging has relatively low spatial resolution for the resultant strain maps, and the image analysis of tagging data is time consuming. PC velocity encoding suffers from errors accumulated across cardiac phases, and complicated tracking and integral algorithms have to be applied to correct these errors to calculate strain rate. Both MR imaging modalities suffer from low blood-myocardium image contrast. Balanced SSFP is an imaging technique that consists of high signal efficiency and is characterized by a strong blood-myocardium contrast. SSFP is used to quantify global parameters such as ejection fraction and mass, however, SSFP endures off resonance effects. A commonality between all techniques lies in the essential step of demarcating the left ventricle (LV) from surrounding structures during CMR image analysis. However, the anatomical nature of the heart and limitations in CMR techniques can make it difficult to distinguish LV boundaries. Extensive research has been dedicated to formulating various semi-automated and automated methods for segmenting the myocardium in CMR images. In myocardial tagging, segmentation methods include deformable models [7,8] and a combination of active contours and region based segmentation techniques [9]. Guttman et al. used a dynamic programming method based on a minimum cost algorithm [10], while Alatter et al. incorporated a region growing algorithm [11]. A model based approach is often used in the analysis of LV function and segmentation. Montillo et al. [12] and Young et al. [13] described fully automated and semi-automated segmentation methods [12,13], respectively, using an LV finite element model. In PC velocity encoding CMR, active contour models and the velocity phase data are used to distinguish between myocardium and blood [14]. Kainmüller et al. introduced a method that uses edge detection, curvature, flow and prior shape information [15]. Techniques for segmenting SSFP images mostly incorporate image processing methods, which include thresholding, edge detection, mathematical morphology, and image filtering [16]. Prior geometric and spatiotemporal information methods are described in [17,18].

Displacement encoding with stimulated echoes (DENSE) is a CMR technique well suited to quantifying regional functional parameters of the heart. This dark blood imaging technique provides regional myocardial displacement and strain measurements. The tissue displacement is encoded directly into the phase of the stimulated echo (typically with reference to end diastole), allowing for the extraction of motion and strain data at a pixel resolution [19,20]. DENSE benefits from the advantages of both myocardial tagging and PC velocity encoding, and is capable of measuring large displacements over reasonable periods of time at a high spatial resolution. A free-breathing navigator-gated 3D spiral cine DENSE sequence has been developed to quantify tissue motion and strain within the entire LV in a single scan [21]. Like the other methods, DENSE ventricular analysis requires LV myocardial segmentation from surrounding structures. However, there are fewer LV segmentation algorithms for DENSE data analysis. Spottiswoode et al. presented a 2D semi-automatic segmentation algorithm guided by the phase information inherent in the DENSE data [22]. Chen et al. further described a method to segment myocardial contours using image intensity standardization and model evolution techniques [23]. However, to date, the segmentation processes in most reported studies were usually performed manually by an experienced user [21,24]. A 3D cine DENSE data set typically comprises over 600 epicardial and endocardial LV contours, which would take an experienced user between 1 and 2 hours to demarcate. The clinical implementation of this technique is therefore limited by this necessary but prohibitively time consuming step. The purpose of this study was to develop a tailored semi-automated segmentation algorithm for 3D cine DENSE CMR. This algorithm is largely based on the DENSE phase images, allowing 3D tissue tracking methods and an LV finite element model to drive the segmentation across the cardiac cycle. To date, there is no universal acceptable segmentation method which can produce satisfactory results in a broad range of cardiac imaging processing applications. Most algorithms consist of limitations and assumptions based on imaging methods and data acquired. Evaluation of segmentation results can therefore be a challenging task [25].

This work presents a semi-automated segmentation algorithm for 3D cine DENSE MR LV data. A finite element model is used to capture the volumetric epicardial and endocardial surfaces, and propagate each surface across the cardiac cycle using the inherent displacement properties found in DENSE MR data. Epicardial and endocardial contours are extracted at each relevant cardiac time point. The time for the segmentation process is significantly reduced and the methods presented allow for the first cardiac frame to be demarcated, where the blood-myocardium contrast is low. This algorithm’s results are evaluated by assessing the algorithms accuracy, precision and efficiency.

## Methods

### Data acquisition

Three dimensional cine DENSE data of the whole heart was acquired for four healthy male volunteers (age range 21 – 45) on a 1.5 T CMR scanner (Siemens MAGNETOM Avanto, Erlangen, Germany) using a six channel phased-array radio frequency (RF) coil. The entire heart was imaged in short axis slices at a 2.8 × 2.8 × 10 mm^3^ spatial resolution and 32 ms temporal resolution. Fourteen 3D partitions were acquired. Zero-padding was used during image reconstruction to increase the 14 acquired 3D partitions to 28 partitions, reducing the through plane resolution to 5 mm. Three partitions at each end of the volume were discarded after the Fourier Transform in the partition direction was applied. This resulted in a 3D image matrix size of 128 × 128 × 22. Displacement was encoded in three orthogonal directions. Images were acquired during a 20.5 ± 5.7 min scan with prospective ECG cardiac gating and diaphragmatic navigator respiratory gating. Other imaging parameters include: field of view (FOV) = 360 × 360 × 140 mm^3^, displacement encoding frequency k_e_ = 0.06 cycles/mm, ramped flip angle up to 20 degrees, TR = 16 ms, TE = 1.3 ms. A spiral readout was used with 6 spatial interleaves per 3D partition. Spiral aliasing artifacts existed on the edges of images, however, were not present on the heart, which was placed at the center of the FOV. All imaging was conducted with informed consent and IRB approval.

### Image analysis

**Figure 1:**
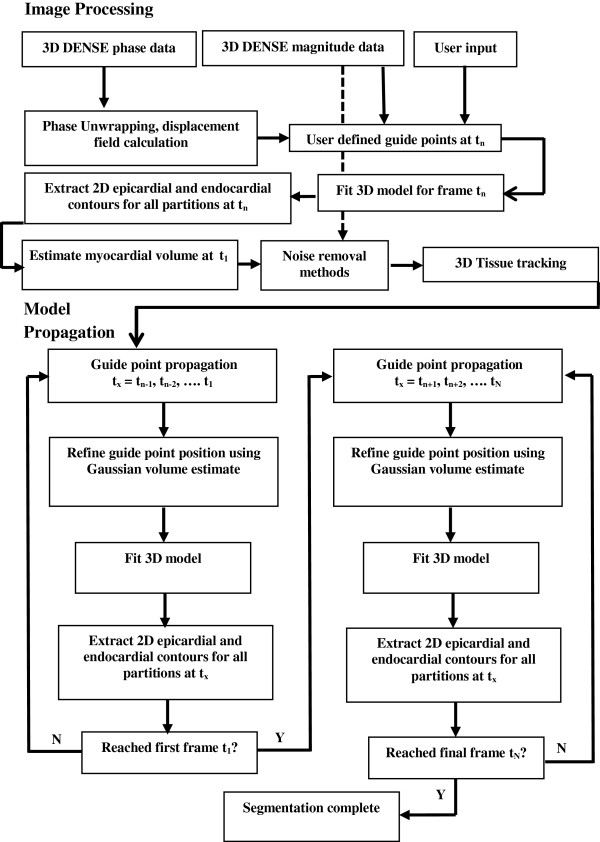
**Image processing and model propagation.** Flow diagram shows each step from image processing, definition of guide points, and model propagation through the cardiac cycle, required for the segmentation algorithm.

Each step in the segmentation algorithm is described below, while a summary of the algorithm is shown in Figure 1. All software development was performed using MATLAB (The Mathworks, Natick, MA). All computation was completed on an Intel(R) Core(TM) i3 CPU, at 2.27 GHz with 4GB of RAM.

### Phase unwrapping and estimation of displacement fields

**Figure 2:**
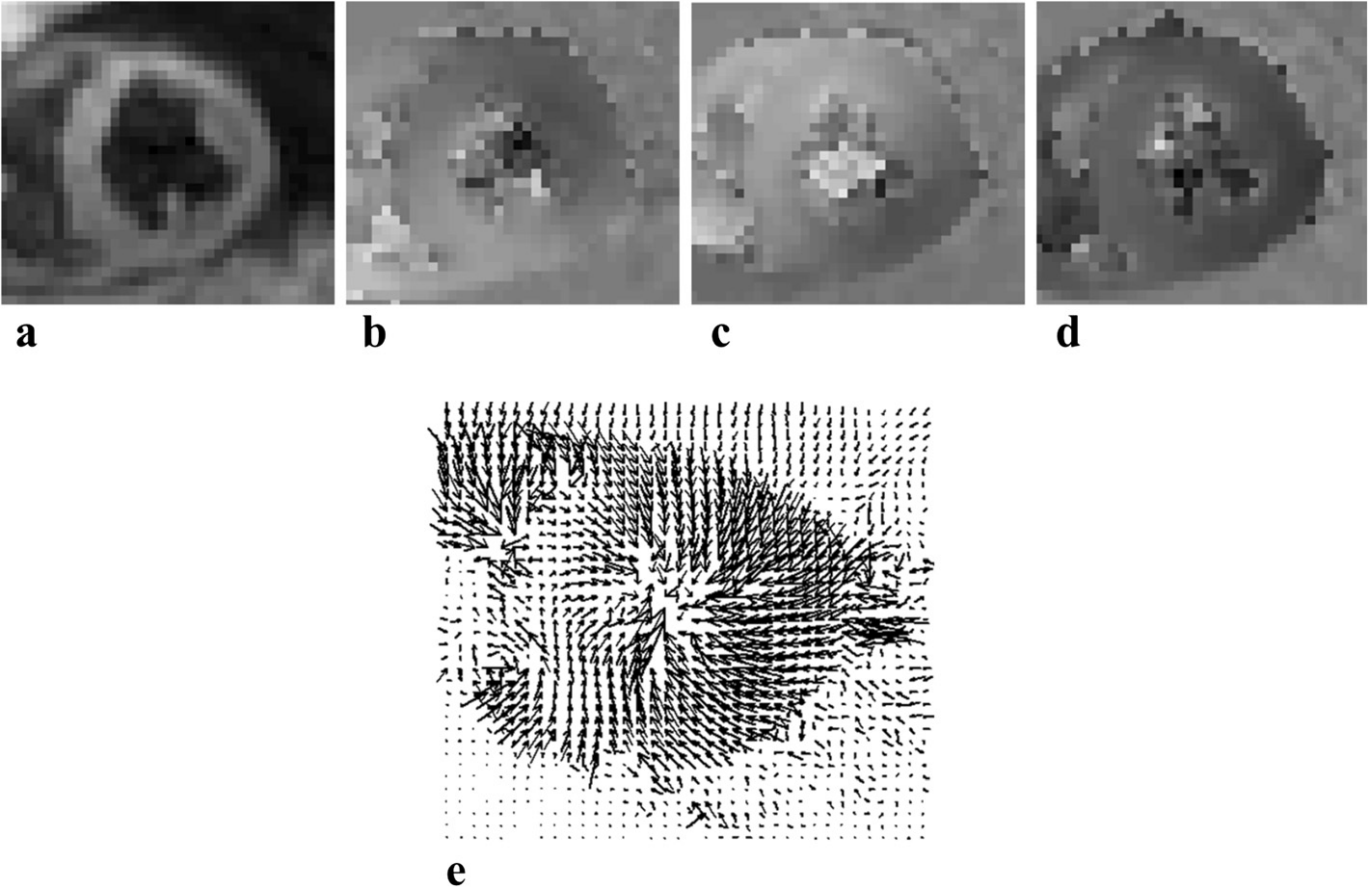
**A single short axis slice of the 3D cine DENSE data during systole. (a)** DENSE magnitude image, **(b, c, d)** DENSE phase images after applying spatiotemporal phase unwrapping without predefined contours, encoded for motion in the x, y and z directions, respectively. **(e)** Corresponding 3D DENSE displacement field derived (view from above).

Spatio-temporal phase unwrapping [26] was performed in order to remove phase aliasing and acquire the absolute displacement measurements. The phase data from each of the three encoding directions were combined to create 3D Eulerian displacement fields. Figure 2 illustrates un-contoured cine DENSE magnitude and unwrapped phase images in three orthogonal directions, with the corresponding 3D displacement field (view from above) for a single short-axis slice. The LV displacement vectors are evident as continuity in the image, illustrating the contracting motion of the LV, as the displacement vectors are pointing towards the center of the LV. In contrast, displacement vectors describing the surrounding tissue such as the liver are coherent in a single direction, while the lungs and blood are represented by randomly scaled and oriented vectors.

### Initializing epicardial and endocardial surfaces

**Figure 3:**
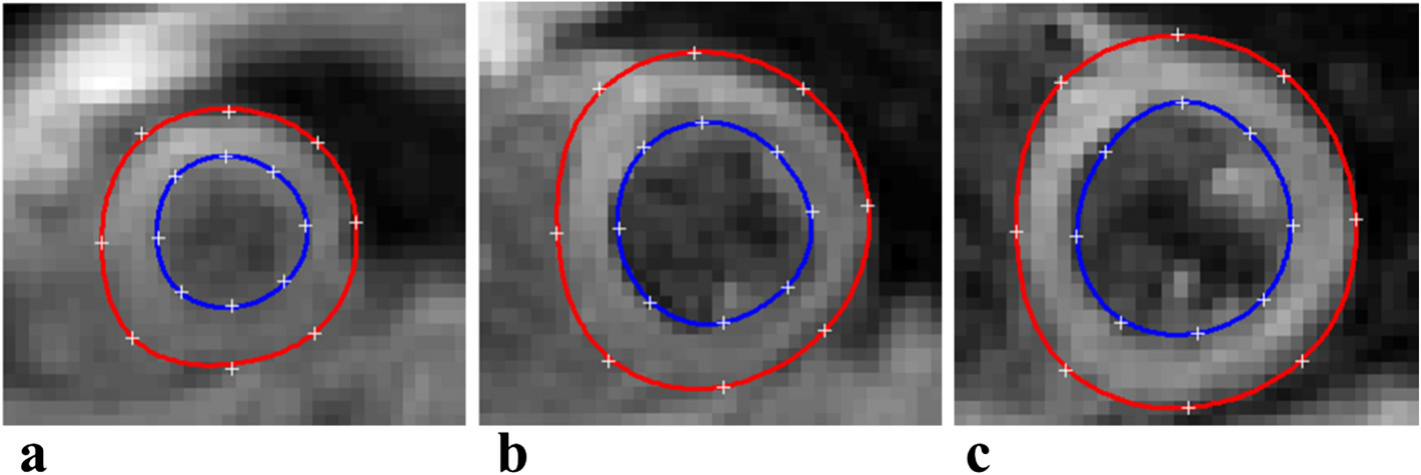
**3D DENSE magnitude images at (a) apex, (b) mid LV and (c) base.** Each image shows 8 spatially placed user defined guide points (+) at the epicardial and endocardial boundaries, with the corresponding LV model defined epicardial (red) and endocardial (blue) contours.

A 3D finite element model of the LV was employed to create each epicardial and endocardial surface separately [27,28]. Each surface is manually initialized at a single time point by defining 3 sets of coordinates. First, the user defines points along the corresponding border at the most apical and the most basal slice. The last coordinate required corresponds to the position of the mid LV – mid septum. The surface is then defined by interactively placing guide points along the respective boundary in a Cartesian coordinate system. This is done on the DENSE magnitude images of all the slices at any single cardiac time point (t_n_) where the LV myocardium is easily distinguishable from the surrounding structures. Each 3D surface point is transformed into a prolate spheroidal coordinate system as described in [27]. Prolate spheroidal coordinates provide a convenient representation of the LV geometry, and further facilitate surface calculations in a deforming LV. Figure 3 illustrates the user defined guide points placed at three different short axis slices (apical, mid and basal) at each LV boundary during a systolic cardiac time frame. During initialization, each surface consists of a number of parameters which can affect the accuracy of the model. These parameters include the number of user defined guide points, the magnitude of the smoothing weight constraints, and the model mesh size which is dependent on the number of elements. In this work, we define a set of initial model parameters using 8 guide points to define each surface, yielding an ellipsoidal mesh composed of 32 bicubic Hermite elements (4 circumferential and 8 longitudinal). Each surface was defined at an early systolic time frame (t_n_), with smoothing weights α and β consisting of values of 0.1 × 10^-1^ and 0.2 × 10^-1^ respectively. The ratio of the smoothing weights (α/β) penalizing bending and stretching of the fitted surface, is maintained at 0.5 [27,28]. Hashima et al. describes the implementation of weight constraints and its effects [28].

The model describes the complete 3D LV epicardial and endocardial surface at the cardiac frame, t_n_. Each surface is then sampled at the z-coordinate corresponding to the data slice in order to extract a set of initial 2D contours.

### Noise removal and tissue tracking

In DENSE, the displacement vectors at all-time points in the cardiac cycle will reference the material position of the myocardium at the time of encoding. This inherent property of DENSE, allows for the myocardium enclosed in the first set of contours derived from the user defined guide points, to be estimated at the first time frame (t_1_), where tissue tracking is initiated. Obtaining suitable motion trajectories from noisy displacement vector fields requires tissue tracking refining steps where unwanted vectors are excluded from tissue tracking.

A spatial derivative function and a low signal-to-noise (SNR) filtering step were implemented in order to remove the effects of noisy vectors from the lungs and blood pools, and include vectors only corresponding to the LV myocardium in the tissue tracking methods. This is a two-step process where a modulus deformation mask is applied prior to tracking where the majority of unwanted vectors are removed, followed by an SNR filter is then applied to further exclude any further noise surrounding the LV.

Combining the orthogonal spatial derivatives of the displacement fields at each cardiac time point creates a modulus deformation mask [22]. A threshold of 80% is used to exclude noisy vectors. This limit is well above myocardial deformation values found in the healthy LV [29]. The spatial derivative for a single time point (t_n_) is given by Equation 1.

(1) $$\begin{array}{ll} S\left(X,t_{n}\right)= & \frac{1}{2}\sum_{k=\hat{i},\hat{j},\hat{k}}\;\left[{\left(\frac{\partial u_{k}\left(X,t_{n}\right)}{\partial \hat{i}}\right)}^{2}+{\left(\frac{\partial u_{k}\left(X,t_{n}\right)}{\partial \hat{j}}\right)}^{2}\right)\\ & {\left(+{\left(\frac{\partial u_{k}\left(X,t_{n}\right)}{\partial \hat{k}}\right)}^{2}\right]}^{\frac{1}{2}} \end{array}$$

where *X* represents the spatial position in the image plane, $u_{\hat{i}}$, $u_{\hat{j}}$ and $u_{\hat{k}}$ represent the displacement vector components in the three orthogonal directions, $\hat{i}$, $\hat{j}$, and $\hat{k}$, respectively.

An SNR filter is implemented in order to remove low SNR voxels surrounding the LV. The filter is based on the myocardial image intensity and the background noise. A region of background noise is identified by the user, and the myocardium intensity is defined by the tissue enclosed within first set of contours initiated at time t_n_ at each slice, for all slices. The mean and standard deviation of the enclosed myocardium is calculated and voxels below two standard deviations are excluded.

Previous DENSE tracking algorithms describe the interpolation of vector displacement fields for tissue tracking through the cardiac cycle [24,26]. This work implements a similar tracking algorithm, where the position of each tissue tracking point along its motion trajectory was estimated using 3D distance weighted linear interpolation, using the entire 3D displacement vector volume. Trajectories are further improved by applying temporal fitting for each ordinate direction of each of the trajectories using a 10^th^ order polynomial [21].

Using 3D motion trajectories, each user defined guide point can be propagated to successive cardiac frames. The time direction in which this is done is irrelevant.

### Guide point propagation

**Figure 4:**
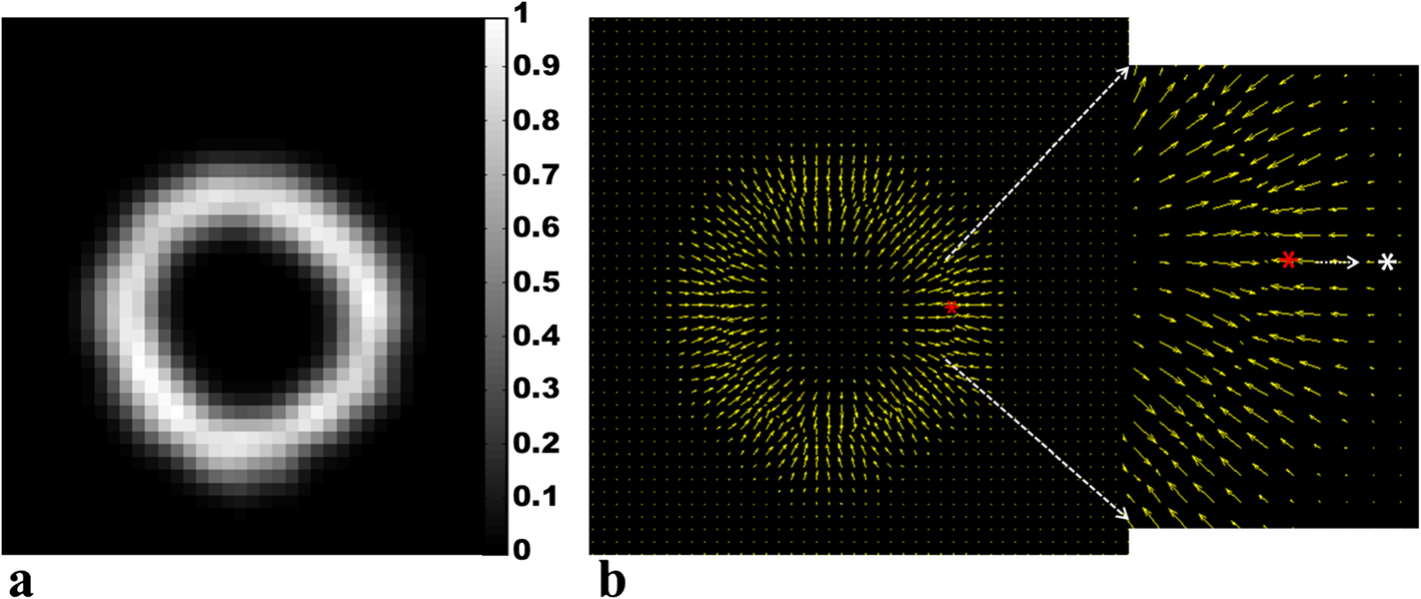
**Propagated guide point refinement step. (a)** Gaussian image with an integrated intensity of unity at a systolic time frame. **(b)** Vector field corresponding to the Gaussian intensity gradient. The red star represents a user defined epicardial guide point which has been tracked to this time frame using 3D motion trajectories. The gradient vectors are used to drive this point to the epicardial boundary to a Gaussian intensity value of 0.5. This is represented by the white arrow. The new guide point for this time frame is thus represented by the white star.

The position of the guide points at all time frames in the cardiac cycle can be estimated using the closest myocardial motion trajectory to each guide point. A refinement step is necessary to ensure that the guide point position accurately coincides with the border of the myocardium. This is achieved using a separate series of images, which are created by representing each trajectory position at each frame by a 3D Gaussian function with an integrated intensity of unity. The contribution from all tracked points is added to create a combined Gaussian image at each time frame. This is illustrated in Figure 4(a). As each guide point is propagated across the cardiac cycle, its position is refined by moving the point along the intensity gradient of the combined Gaussian image towards the endocardial/epicardial boundary until an intensity value of 0.5 is reached. This is illustrated in Figure 4 (b). A Gaussian standard deviation of 1.25 was used for the in-plane *x* and *y* directions, while a standard deviation of 1.5 was used in the *z* direction allowing for an ellipsoidal distribution. This was found suitable as the slice thickness dimension is greater than the in-plane resolution. If the standard deviation is set too low, there is a pixelated effect in the Gaussian image where the distribution is unequal, while if the standard deviation is set too high the distribution is expanded and the LV dimensions are shown to be much larger, similar to the effect described in [22].

### Experimental validation

#### Accuracy

The accuracy of the algorithm is assessed by the degree to which the segmentation results compare to a reference standard. In order to quantify the voxel-based accuracy of the segmentation results to a reference standard, all four 3D data sets were manually contoured by a single experienced user. The semi-automatic segmentation was completed for all four data sets using the model parameters described above which were chosen based on a visual assessment of accuracy. The following model parameters were then individually varied in order to determine the optimal parameters and how each can have an effect on the segmentation results: (1) the model was initialized during a late systolic time frame, as opposed to an early systolic time frame, (2) four user defined guide points defined each surface, as opposed to the initial eight, (3) the smooth weight constraints α and β were decreased from 0.1 × 10^-1^ and 0.2 × 10^-1^ to 0.1 × 10^-3^ and 0.2 × 10^-3^, respectively, and (4) the model mesh size was adjusted from 32 to 64 elements. The accuracy of the algorithm was assessed using the following two methods:

1. To quantify the accuracy of the 2D contour geometry, three slices (apical, mid and basal) at end diastole and end systole were manually contoured by 2 separate operators (Operator A and Operator B). The corresponding semi-automated contours were validated against each of these manual contours by comparing the percentage of overlapping and discrepant voxels with the use of region-based coefficients. This was done by calculating mean and union overlaps (Dice and Jaccard metrics) [25,30]. A value of zero indicates no spatial overlap, while a value of 1 indicates complete perfect overlap. The Dice (D) and Jaccard (J) coefficients are calculated by D = 2((S_r_ ∩ T_r_)/S_r_ + T_r_) and J = ((S_r_ ∩ T_r_)/S_r_ ∪ T_r_), respectively. Here, S_r_ is the region on the source image enclosed by the manual contour and T_r_ is the region on the target image enclosed by the semi-automated contour. ∩ and ∪ denote intersection and union, respectively. A value of 0.7 and above is considered an adequate overlap [30].

2. The automatic contours where further compared to the manual contours of each operator using false positive and false negative area measures. The false positive measure indicates the percentage of tissue that is falsely identified as myocardium, while false negative measures indicate the percentage of myocardium missed by the algorithm. The false negative error is given by (T_r_ - (S_r_ ∩   T_r_))/S_r_ and the false positive error is given by (S_r_ – (S_r_ ∩ T_r_))/S_r_.

#### Precision

Precision refers to the repeatability of the segmentation algorithm when applied to the same data set. This is accomplished by applying the segmentation process to the same data set a number of times and asses the results. The algorithm was applied 5 times at the same systolic time frame to two arbitrarily selected data sets. An arbitrary slice at a specific cardiac phase was selected and the Dice, Jaccard, false positive and false negative measures were calculated by comparing the model derived contour to a contour manually delineated by a single operator.

#### Efficiency

As described above, the segmentation process is the most time consuming and limiting step in cardiac MR analysis. The efficiency of the algorithm provides the information on the practical use of the algorithm, computational complexity and processing time. The timing of the algorithm is divided into three parts: model initialization, 3D tracking with temporal fitting and surface model propagation.

## Results

**Figure 5:**
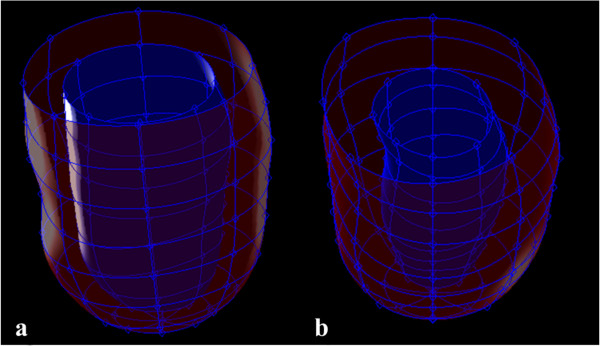
**Three dimensional LV model epicardial (red) and endocardial (blue) surface as a result of user defined guide points.** Surface shown during, **(a)** end-diastole, and **(b)** end-systole.

The complete semi-automated segmentation process takes approximately 10–15 minutes per subject which includes phase unwrapping and displacement field calculations. The segmentation algorithm’s time is divided between three stages: manual guide point placement (~4 minutes), 3D tracking and temporal fitting (~ 3 min) and the model propagation time (~3 - 4 minutes). Figure 5 (a,b) illustrates examples of epicardial and endocardial generated surfaces, where (a) represents a case where both surfaces were at an end diastolic time frame, and (b) represents a case where both surfaces were at end systole.

**Figure 6:**
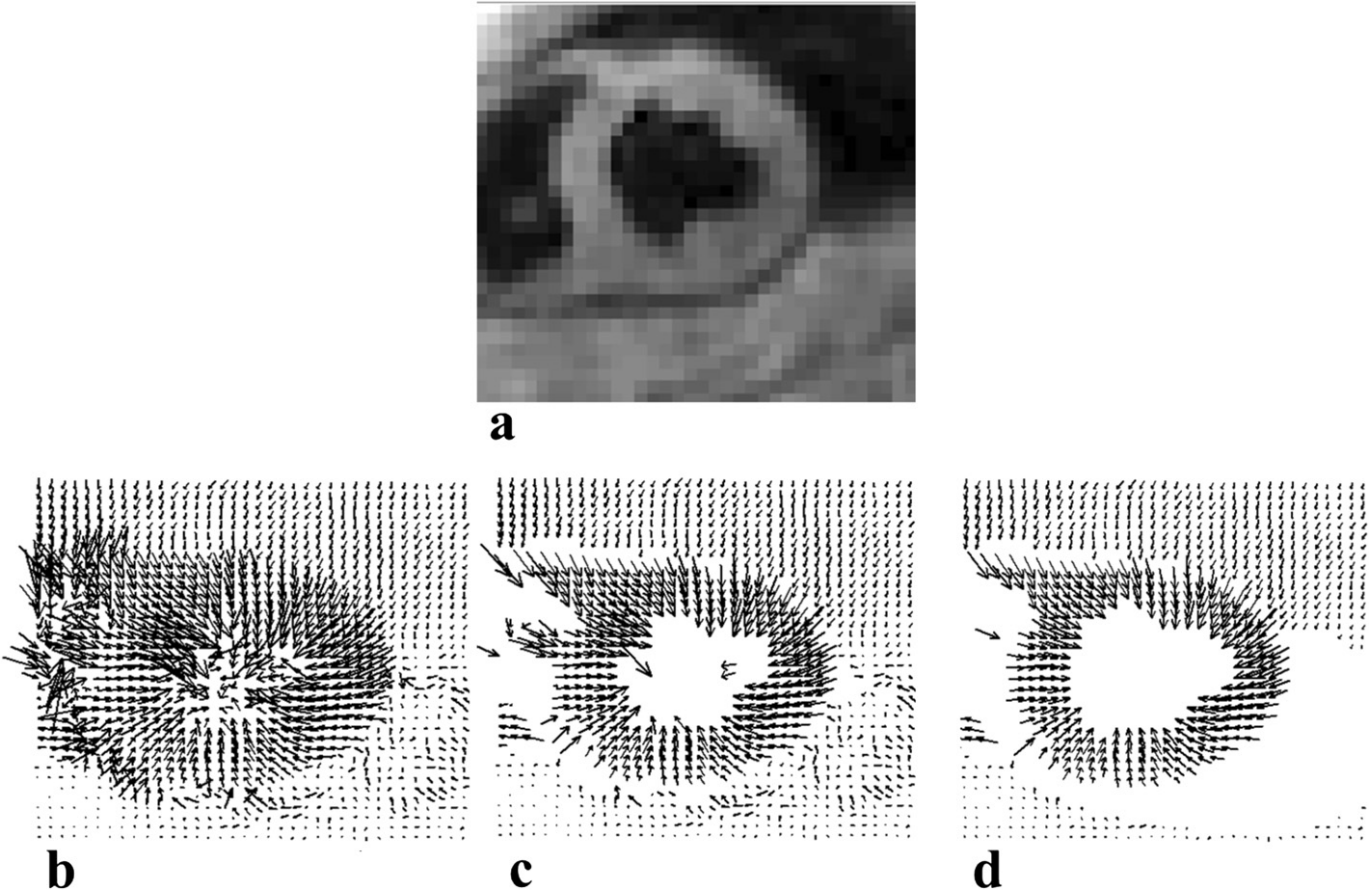
**Noise removal methods. (a)** LV-mid DENSE magnitude image during end-systole, **(b)** corresponding vector displacement field without pre-defined contours, **(c)** vector displacement field after applying modulus deformation mask, showing removal of randomly orientated vectors predominantly in the blood pool, **(d)** vector displacement field after applying the SNR filter, further removing noisy vectors. Vectors incorporated in 3D tissue tracking.

Figure 6 illustrates, for a selected short axis slice, the effects of the noise removal techniques used to discard unwanted displacement vectors. These images show how each step removes randomly oriented and scaled vectors, while the vectors describing myocardium remain.

**Figure 7:**
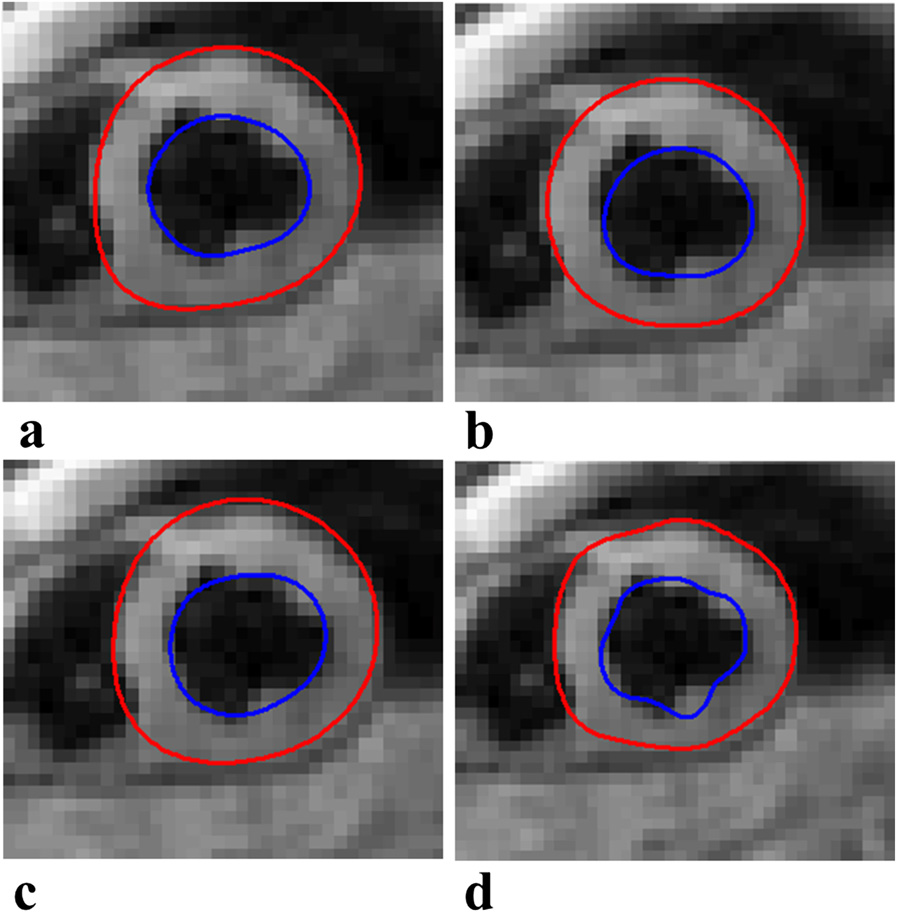
**Segmentation results during end-systole.** Each image represents the results after varying an individual model parameter. **(a)** Initialization of model during end-systole as oppose to early-systole, **(b)** decreased user guide points from 8 to 4 per short axis slice, **(c)** model mesh size is increased from 32 to 64 elements and **(d)** smoothing weight factors reduced from α = 0.1 × 10^-1^ to 0.1 × 10^-3^ and β = 0.2 × 10^-1^ to 0.2 × 10^-3^.

Figure 7 illustrates contoured data of a mid-ventricular slice during end-systole for varying model parameters: (a) *corresponds to initializing the model during end-systole*, 8 guide points per slice, model mesh size of 32 elements and smoothing weight factor of α = 0.1 × 10^-1^ and β = 0.2 × 10^-1^, (b) corresponds to initializing the model during early-systole, *4 guide points per slice*, model mesh size of 32 elements and smoothing weight factor of α = 0.1 × 10^-1^ and β = 0.2 × 10^-1^, (c) corresponds to initializing the model during early-systole, 8 guide points per slice, *model mesh size of 64 elements* and smoothing weight factor of α = 0.1 × 10^-1^ and β = 0.2 × 10^-1^ and (d) corresponds to initializing the model during early-systole, 8 guide points per slice, model mesh size of 32 elements and *reduced smoothing weight factors* of α = 0.1 × 10^-3^ and β = 0.2 × 10^-3^.

A summary of the Dice and Jaccard spatial overlap, false positive and false negative results between two observers and the model is given in Table 1. Table 2 shows the spatial overlap results between a single observer and the model at an early systolic time frame, given individual parameter variation.

**Table 1:** Spatial overlap results between contours defined by 2 separate operators (A and B) and the model derived contours at an early systolic time frame

**Measure**	**Operator A vs. model**			**Operator B vs. model**			**Operator A vs. B**		
	***Apex***	***Mid***	***Base***	***Apex***	***Mid***	***Base***	***Apex***	***Mid***	***Base***
Dice coefficient	0.85±	0.87±	0.88±	0.86±	0.89±	0.85±	0.88±	0.88±	0.92±
	0.06	0.04	0.02	0.02	0.03	0.03	0.04	0.02	0.03
Jaccard coefficient	0.75±	0.77±	0.78±	0.76±	0.80±	0.73±	0.79±	0.78±	0.86±
	0.09	0.06	0.03	0.02	0.04	0.05	0.06	0.04	0.05
False positive	0.09±	0.11±	0.08±	0.06±	0.09±	0.1±	0.16±	0.12±	0.03±
	0.1	0.02	0.07	0.04	0.03	0.08	0.07	0.06	0.04
False negative	0.19±	0.14±	0.16±	0.19±	0.13±	0.19±	0.09±	0.13±	0.11±
	0.06	0.06	0.08	0.02	0.06	0.08	0.02	0.06	0.04

The model here used the initially defined parameters.

**Table 2:** Contour overlap validation between a single operator and geometrical model with individual parameter variation

	**4 Guide points**			**64 Model elements**			**Late systolic initialization**			**α = 0.1 × 10**^**-3 **^**β = 0,2 × 10**^**-3**^		
	***Apex***	***Mid***	***Base***	***Apex***	***Mid***	***Base***	***Apex***	***Mid***	***Base***	***Apex***	***Mid***	***Base***
Dice coefficient	0.85±	0.86±	0.86±	0.80±	0.85±	0.85±	0.89±	0.92±	0.87±	0.81±	0.88±	0.86±
	0.03	0.04	0.05	0.14	0.03	0.08	0.03	0.87	0.15	0.03	0.01	0.03
Jaccard coefficient	0.74±	0.76±	0.76±	0.68±	0.73±	0.75±	0.81±	0.85±	0.79±	0.68±	0.78±	0.76±
	0.04	0.07	0.08	0.17	0.05	0.11	0.05	0.02	0.21	0.04	0.02	0.05
False positive	0.05±	0.07±	0.06±	0.08±	0.09±	0.05±	0.03±	0.08±	0.04±	0.04±	0.10±	0.08±
	0.04	0.04	0.03	0.01	0.05	0.03	0.03	0.02	0.02	0.04	0.08	0.02
False negative	0.22±	0.18±	0.19±	0.26±	0.19±	0.21±	0.17±	0.08±	0.18±	0.28±	0.14±	0.18±
	0.05	0.10	0.09	0.19	0.09	0.10	0.06	0.03	0.21	0.06	0.07	0.06

The segmentation process was run five times for two separate data sets. Table 3 illustrates Dice, Jaccard, false positive and false negative measurements for each data set.

**Table 3:** Spatial overlap results demonstrating the algorithm’s precision. The algorithm was run five times on two arbitrarily selected data sets

	**Dice**		**Jaccard**		**False positive**		**False negative**	
**Iteration**	**Set 1**	**Set 2**	**Set 1**	**Set 2**	**Set 1**	**Set 2**	**Set 1**	**Set 2**
1	0.9225	0.9083	0.8562	0.8321	0.0162	0.0856	0.1299	0.0586
2	0.9064	0.9187	0.8289	0.8496	0.0608	0.0776	0.1331	0.0628
3	0.9068	0.9160	0.8295	0.8450	0.0481	0.0972	0.1306	0.0729
4	0.9183	0.9261	0.8490	0.8623	0.0719	0.0833	0.0899	0.0658
5	0.9115	0.9370	0.8374	0.8814	0.0656	0.0202	0.1158	0.1008

An arbitrary slice at an early systolic cardiac phase is selected and was manually contoured by a single Operator. The spatial overlap measures were calculated after each experiment. The results for each data set show the consistency of the algorithm.

**Figure 8:**
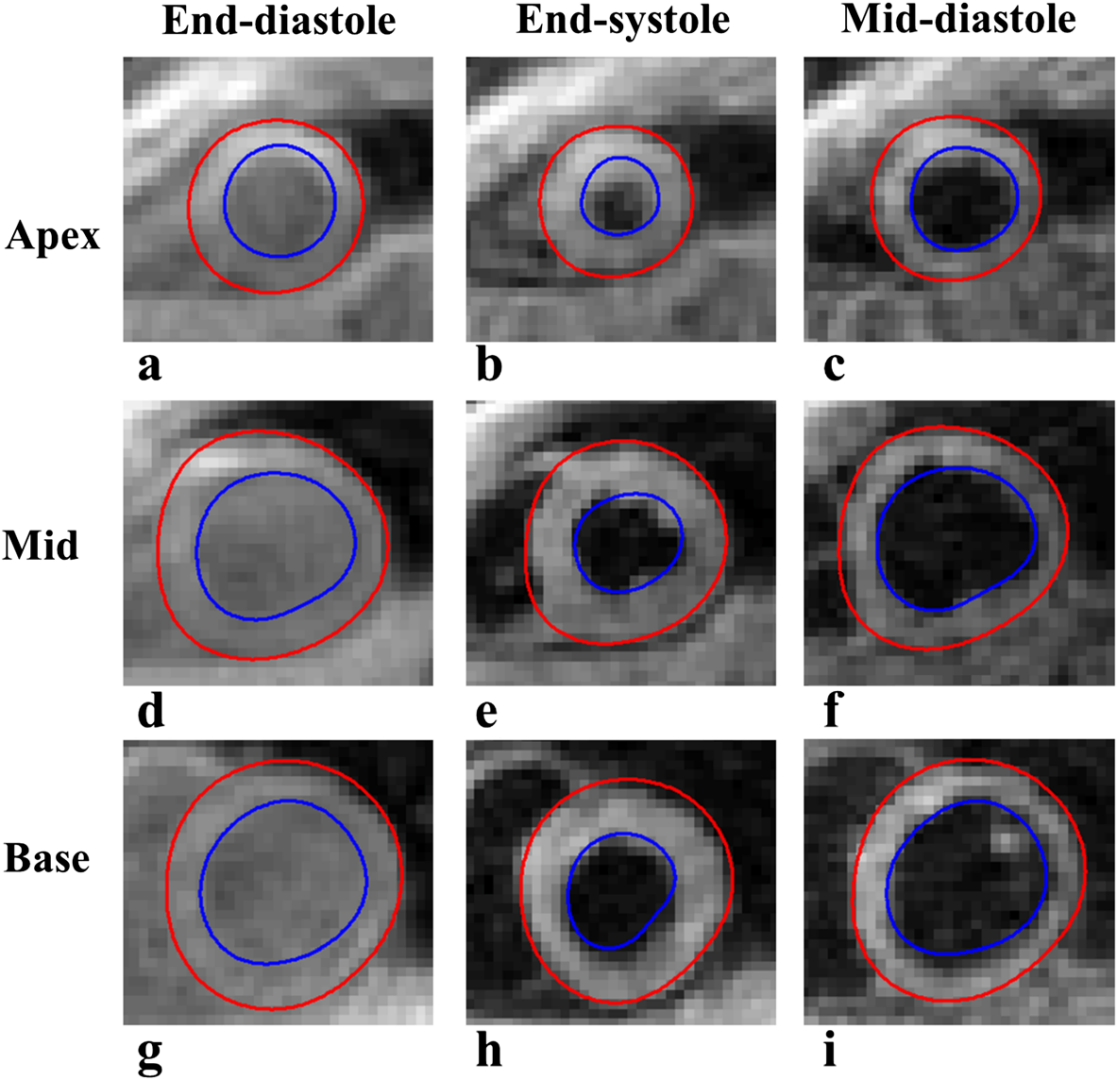
**Segmentation results according to the initial LV model parameters: 8 user defined guide points, 32 element model defined at early-systole, with smoothing constraints of α = 0.1 × 10**^**-1**^** and β = 0.2 × 10**^**-1**^**.** The results are shown at the LV apex **(a, b, c)**, mid **(d, e, f)** and base **(g, h, i)** at three separate cardiac time frames; end-diastole **(a, d, g)**, end-systole **(b, e, h)** and mid-diastole **(c, f, i)**.

Figure 8 (a - i) illustrates the segmentation results during three stages of the cardiac cycle, at an apical, mid and basal slice. The results are based on the initial chosen model parameters. Figure 8 (a,d and g) shows the algorithm’s ability to enclose myocardium during end diastole, where it is visually difficult to discern between the myocardium and blood pool. Furthermore, all images show a visual accurate propagation of each surface across the cardiac cycle, by enclosing the LV myocardium and the three distinct time frames. Based on a visual analysis by an experienced user, only 125 out of a total of 2124 contours from all 4 data sets, or 5.6%, would require readjustment.

## Discussion

This study presents a semi-automated LV segmentation algorithm for 3D cine DENSE CMR, using a guide point model approach. An LV finite element model has been used for segmentation with other 3D CMR studies [13,31-33]. However, in previous work the model is typically manually initialized at each cardiac frame, whereas here the information inherent in the DENSE phase images provides the ability to propagate the model across the cardiac cycle. The use of guide points reduces the user interaction time to manually demarcate the LV. Furthermore, as the algorithm allows for the user to define guide points at any cardiac frame where the myocardium is clear and distinguishable, the risk of user input error is reduced. The model is sufficiently deformable to adhere to variations in LV shape and consists of smoothing constraints to correct for a degree of guide point misalignment [27,28].

The time required to manually contour an entire 3D DENSE data set for an experienced user is approximately 1–2 hours, whereas this semi-automated algorithm reduces the process to approximately 15 minutes. This time could be further reduced by altering model parameters such as the number of guide points used and the mesh size, but at the expense of segmentation accuracy. Computation time can further be improved by implementing the algorithm in a more efficient programming language (such as C++), and upgrading the computer hardware specifications.

As demonstrated by Figure 6, methods used to remove noisy vectors are shown to work appropriately, limiting the displacement fields predominantly to myocardial vectors. The spatial derivatives remove the majority of unwanted vectors by providing a deformation mask around the LV. As 3D spiral cine DENSE boasts a higher SNR than echo planar approaches [21] and a varying flip angle is used, an SNR threshold is appropriate to further remove unwanted vectors in the lungs and blood pools.

Tissue tracking using a full 3D volumetric displacement field at each frame, will improve the tracking accuracy in the LV compared to 2D DENSE.

Table 1 shows the results of the spatial overlap results in the form of Dice and Jaccard coefficients, and false positive and false negative overlap results. The results were compared between the contours drawn by two separate Operators (A and B), and the initial model parameter derived contours. According to Zou et al. [30], Dice and Jaccard coefficient value of 0.7 and above, indicates a good spatial overlap. All Dice and Jaccard coefficient results shown are well above 0.7, except for two measurements, 0.68 ± 0.17 and 0.68 ± 0.04 using a 64 element model and half the smoothing factor respectively. Both results correspond to values at the LV apex, where the myocardial contrast is lower and is the area with the greatest curvature.

The highest Dice and Jaccard coefficient values were between Operator A and B, but the variations between each operator and the model were insignificant, implying a good spatial overlap agreement between manual contours from the two separate operators, and the model defined contours.

A low false positive signifies a low percentage of tissue falsely identified as myocardium and a low false negative result signifies a low percentage of missed myocardium. Interestingly, the highest false positive values occurred between operator A and B, with values of 0.16 ± 0.07 and 0.12 ± 0.06 for the apex and mid LV respectively. This could be attributed to the operators having inconsistent estimation of papillary muscles and the perimeter of the endocardium at the apex of the LV. Although the majority of the blood has been expelled, the contrast towards the LV apex is not always as clear as the contrast towards mid and basal slices. It can therefore be argued that when segmenting areas of poor contrast, the algorithm is more reliable than manual methods. False negative results show higher values in the base and apex. At the apex, this could be attributed to the low LV contrast, while the propagation of guide points towards basal LV can produce underestimated epicardial contours due to the high displacement and motion at the base.

As each guide point governs the direction and shape of the surface, using fewer guide points can result in the underestimation of the LV curvature. This is shown by the slightly higher false negative results in Table 2 when using four guide points to initialize each surface. However, the overall spatial overlap results in Table 1 show Dice and Jaccard values well above 0.7, therefore using 4 guide points placed symmetrically around the LV in order to reduce computation time and user interaction, is therefore a reasonable adjustment.

Increasing the model mesh size will increase the computation time, and maintain a more uniform cylindrical surface. In Table 2, Dice and Jaccard coefficients show a good spatial overlap. The higher false negative value at the apex can be attributed to the model’s uniform shape therefore over estimating the endocardial border. The effect of initializing the segmentation process at late systole compared to early systole is summarized in Table 2, where a good spatial overlap, with low false positive values is seen. Slightly higher false negative values are found at the apex and base. The low LV contrast and through plane motion may account for these results, respectively. These results confirm that model initialization can be done at any time frame.

Significantly reducing the smoothing constraints in the model is shown to negatively affect the segmentation results. If a guide point is propagated incorrectly due to phase noise, the ability of the model to correct this misalignment is reduced. In order to maintain accurate contour results while lowering the smoothing constraints, one would have to increase the model mesh size and the number of user defined guide points.

The best model parameters to use in the segmentation based on accuracy, computation time and user required input are as follows; initialize the model at an early systolic frame using 8 guide points to define each surface, yielding an ellipsoidal mesh composed of 32 bicubic Hermite elements. Smoothing weights α and β of values of 0.1 × 10^-1^ and 0.2 × 10^-1^ respectively.

Two data sets were each run through the segmentation process five times. A single slice was arbitrarily chosen at an early systolic cardiac phase. The same slice was contoured at the same cardiac phase by a single operator. The results after each experiment are then examined using Dice, Jaccard, false positive and false negative measures. The results are shown in Table 3 and illustrate the algorithm’s repeatability. There is a clear consistency within each data set for each experiment the algorithm was run.

There are a number of advantages to the semi-automatic segmentation method presented when compared to the current manual methods. The semi-automated technique offers minimal user interaction, a 10-fold reduction in total processing time, and reliable contouring at the first cardiac phase where it can be difficult to visually discern between blood and myocardium.

The method also consists of several limitations and potential sources for error, such as partial volume effects, phase unwrapping errors, and imperfect phase correction. The accuracy of the algorithm therefore fundamentally relies on correct displacement vector calculation, noisy displacement vector removal techniques and accurate tissue tracking. Towards the LV base, the thin myocardial walls, high longitudinal displacements and partial volume effects due to through-plane dephasing can cause inaccurate displacement measurements, causing poor guide point propagation in these areas. The increase of the number of guide points can result in more accurate contours but at the expense of processing time and increased user interaction. However, by adjusting the model parameters accordingly, the work shows reasonably accurate segmentation results with good visual agreement. A current geometrical limitation of this analysis is that the base plane is not incorporated in the model and tracked through the cardiac cycle. A number of basal slices are thus removed to avoid the outflow tract moving into the volume of DENSE data being assessed. Finally, although results show good accuracy and repeatability of the algorithm, this work has only been tested on four data sets. Future work must incorporate an increased number of healthy and diseased hearts in order to further validate this algorithm.

## Conclusion

The guide point modelling approach to segment 3D cine DENSE data shows promising results, offering a significant reduction in the segmentation time required for a 3D data set. The DENSE phase data, noise removal methods and 3D tissue tracking techniques allow for a finite element model to be propagated across the cardiac cycle, while adjusting to the geometrical shape of each epicardial and endocardial surface. This allows for the contouring of the entire data set including the first cardiac frame where the myocardium-blood contrast is almost non-existent. A comparison between manually defined contours and the model derived contours using overlap coefficients shows promising results. The initial model parameters provide the best results. However, model parameter variations can be used while still maintaining an accurate LV segmentation. This work is a significant step towards the automation of 3D cine DENSE data analysis.

## Competing interests

XZ and BSS are both employees of Siemens Medical Solutions USA, Inc.

## Authors’ contributions

XZ and FHE developed and implemented the 3D spiral cine DENSE pulse sequence and acquired the data. DAA performed the post-processing software development, with support from BSS and XZ. EMM provided supervision and expert advice. DAA and BSS drafted the manuscript and figures. All authors read and approved the final manuscript.
